# Supplementary material for: On the solutions of fractional differential equations with modified Mittag-Leffler kernel and Dirac Delta function: Analytical results and numerical simulations
Source: PLoS One. 2025 Jun 18;20(6):e0325897. doi: 10.1371/journal.pone.0325897 (PMC12176301; doi:10.1371/journal.pone.0325897)
Supplement: Attachment — Submitted filename: responce.pdf [file pone.0325897.s001.pdf]

Response to Reviewers

January 22, 2025

At first, we would like to express our sincere gratitude to the reviewers for their thoughtful comments and valuable feedback. Below, we provide a point-by-point response to each of the reviewer's comments.
Reviewer 1: Authors examined "Authors examined "On the solutions of fractional differential equations with modified Mittag-Leffler kernel and Dirac Delta function: Analytical results and numerical simulations." The paper has potential to be published in this journal after following major revisions.

1. What are basic gaps which are filled in this research study?
   The modified Atangana-Baleanu derivative of the RL-type is an extension of the Atangana-Baleanu derivative, designed to operate in a broader space. This allows for the solvability of related fractional differential equations without the need for additional, unnecessary conditions, as demonstrated in Section 5.

2. Governing equation are note referenced. Reference each equation properly?
   . Done. Please see the updated version.

3. Please show the novelty and research gap at the end of introduction section by raising some questions which are to be addressed?
   Done. Please see lines 37-44 in Page 2 of the updated version.

1. In the introduction references are little in number, increase it by adding more relevant studies.
   Done. We added 3 more related references to the introduction.

2. Is it the original or relevant topic to the field, whose gaps remain to be filled?
   In the field of fractional calculus an open problem is to identify which fractional operator give the best approximation for a given non-local model. This answer depends on the data we have in our hands for a given model.

3. How is this study compared to the previously published? Which issues are addressed in this which are not in the previously?
   The current study is a continuation of ongoing research focused on extending the definitions of fractional derivatives with nonsingular kernels. The primary issue addressed in

this study is the solvability of related fractional differential equations without the need for additional conditions, making it more suitable for modeling dynamical systems.

4. Is the methodology used here is new developed or the extended the previous one? If it is improved, then what are the basic improvements have been carried out?
   We extend the methodology presented in [7] to obtain new results related to the modified AB-derivative of RL-type and demonstrate the significance of this extension.

5. Try to add some more relevant references to enrich the study literature for clear justification.
   Done. We added 3 more related references to the introduction.

6. Authors are advised to explain the graph with physical reasoning about the parameters and properties in which the variations are being observed.
   We added the physical interpretation of the α parameters in the graphs at the end of Section 4.

7. What is the main question addressed by the research?
   The main question addressed is the solvability of certain fractional differential equations involving the Atangana-Baleanu derivative. For further details, please refer to lines 37-44 in Page 2 of the updated version.

8. What does it add to the subject area compared with other published material?
   We introduce, for the first time, new types of fractional differential operators by extending the Atangana-Baleanu operators to function in wider spaces and under weaker conditions.

9. What specific improvements should the authors consider regarding the methodology? What further controls should be considered?
   As a first step we have developed the mathematical background for the investigated problem. The next step is to find some models which are treated within this approach.

10. Are the conclusions consistent with the evidence and arguments presented and do they address the main question posed?. Improve it.
    We added a sperate conclusion section to address these issues

11. What ranges of all parameters should be given? In the caption mention all of the involved pare metric values for which graphs are plotted, it will help to the reader compare the parametric effects for considered values of the parameters.
    Done. We added the ranges of parameters and edit the captions of the figures. Please refer to the paragraph after equation (4.1).

12. Write the future works in this studies.
    Done. Please see the last paragraph in Section 5.

Reviewer 2: Review Report Title: On the Solutions of Fractional Differential Equations with Modified Mittag-Leffler Kernel and Dirac Delta Function: Analytical Results and Numerical Simulations Overall Assessment
The paper addresses the solutions of fractional differential equations (FDEs) incorporating a modified Mittag-Leffler kernel and the Dirac delta function. This topic is both relevant and timely, as fractional calculus continues to find applications in modeling memory and hereditary effects in various physical systems. The use of the modified Mittag-Leffler kernel and the interplay with the Dirac delta function offer an interesting and novel perspective that could expand the applicability of fractional calculus. While the manuscript contains valuable contributions, there are areas requiring improvement in terms of clarity, organization, and rigor. Below, I provide detailed comments and suggestions.

1. To enhance the clarity and accessibility of the proposed methodology, I suggest including a flowchart that outlines the key steps of the approach.
   It is there, please see lines 46-51 in Page 2 of the updated version.

2. Highlight how the proposed method handles challenges like singularities or boundary conditions.
   Here we introduce new types of fractional differential operators by extending the Atangana-Baleanu operators to function in wider spaces and under weaker conditions. The primary issue addressed in this study is the solvability of related fractional differential equations without the need for additional conditions, making it more suitable for modeling dynamical systems.

3. The manuscript includes only one numerical example, which, while illustrative, limits the depth of validation for the proposed methodology. To strengthen the results and provide a more comprehensive evaluation, I recommend the authors to compare the numerical example with solutions obtained using another established method from the literature, such as finite difference, finite element, or other spectral methods. Highlight the differences in accuracy, convergence rates, and computational efficiency between the proposed method and the chosen benchmark method.
   In this paper, we do not introduce any numerical techniques. In fact, we present the solutions in closed form using the results from Proposition 3.1 and then graph them using Mathematica. However, the development of numerical techniques for related nonlinear fractional differential equations is a topic that can be explored in future works. For further details, see the last few lines of Section 5

4. The authors should consider citing additional relevant works in the field. Specifically
   https://doi.org/10.1088/1402-4896/ac1ccf ;
   https://doi.org/10.1155/2021/6662808;
   https://doi.org/10.1155/2020/1274251
   We cited two of the suggested relevant references. However, for the third one we can't download it to assess whether it relevant or not
